# The Impacts of Climate Change on the Potential Distribution of *Plodia interpunctella* (Hübner) (Lepidoptera: Pyralidae) in China

**DOI:** 10.3390/insects13070636

**Published:** 2022-07-15

**Authors:** Jinyu Zhao, Chengfei Song, Li Ma, Xizhong Yan, Juan Shi, Chi Hao

**Affiliations:** 1 College of Plant Protection, Shanxi Agricultural University, Jinzhong 030801, China; zhaojinyu312@163.com (J.Z.); songcf922@163.com (C.S.); mali890310@126.com (L.M.); yanxizhong80@163.com (X.Y.); 2 Beijing Key Laboratory for Forest Pest Control, Beijing Forestry University, Beijing 100083, China

**Keywords:** climate change, *Plodia interpunctella*, CLIMEX, potential distribution, prediction

## Abstract

**Simple Summary:**

*Plodia interpunctella* (Hübner) is an important grain storage pest in China that is distributed in almost every province and that has caused huge economic losses. In this study, we adjusted the biological parameters of the Indian grain borer and then used the CLIMEX model to predict the detailed potential distribution of *P. interpunctella* in China under current and future conditions. Under historical climatic conditions, the central, northeastern, and southern regions of China are highly suitable habitats for it. Because of temperature change in the future climate, suitable habitats will increase in the eastern part of Qinghai and will decrease in the mid-eastern, northeastern, and southeastern parts of China. This study provides important information for controlling the further spread of the Indian corn borer in China.

**Abstract:**

The Indian meal moth *Plodia interpunctella* (Hübner) (Lepidoptera: Pyralidae) is a notorious stored-grain pest that can be found in most parts of China. The corpses, excretions, and other secretions of *P. interpunctella* larvae cause serious grain pollution, seriously affecting the nutritional and economic value of stored grain in China. To elucidate the potential distribution of *P. interpunctella* in China, we used the CLIMEX 4.0 model to project the potential distribution of the pest using historical climate data (1960–1990) and estimated future climate data (2030, 2050, and 2070). Under the historical climate situation, *P. interpunctella* was distributed in most areas of China, and its highly favorable habitats account for 48.14% of its total potential distribution. Because of temperature change in the future climate, suitable habitats will increase in the eastern part of Qinghai and will decrease in the mid-eastern, northeastern, and southeastern parts of China. Under these scenarios, the area of this pest’s highly favorable habitat will be reduced by 1.24 million km^2^, and its proportion will decrease to about 28.48%. These predicted outcomes will help to distinguish the impact of climate change on the potential distribution of *P. interpunctella*, thereby providing important information to design early forecasting and strategies to prevent pest harm to stored grain.

## 1. Introduction

The Indian meal moth *Plodia interpunctella* (Hübner) is a primary injurious insect to raw and processed stored food products [1,2]. The female moth can oviposit 150–200 individual eggs under suitable conditions, and after hatching, larvae can invade or penetrate various packaging materials and infest food products, making the food inedible [3,4,5]. Because of this, *P. interpunctella* can migrate and spread via infected food goods, which is accelerated due to the development of globalization, leading to it spreading wherever there is permanent human habitation [6,7]. *P. interpunctella* harms more than 170 species in storage, including various grains, dried fruits, pulses, dried vegetables, etc. [8]. After the product is infected, the carcasses, excrements, and other secretions of these pests become mixed with it. The product’s germination rate, nutritional value, quality, and safety level will be seriously reduced; thus, *P. interpunctella* has caused great economic losses in the United States, China, Korea, Ethiopia, and in other countries [9,10,11,12]. Due to it being a generalist feeder with a heterogeneous diet and high ability to damage food products, this pest has become the focus of the world for a long time.

*P. interpunctella* is widely distributed and occurs with high frequency in China, and with the warming climate, it has multiplied in the high-latitude regions of China in Alar, Xinjiang [13,14]. The Intergovernmental Panel on Climate Change—IPCC published its Fifth Assessment Report (AR5), which indicates that global climate has seen an upward trend in each year since the publication of the previous report [15]. Relevant studies have confirmed that since 1960, China’s climate has obviously warmed up, with frequency of heatwaves increasing and glaciers retreating [16]. Climate warming has increased the population growth and metabolic rates of insects, making crop losses more serious [17]. The research on *P. interpunctella* in China mainly focuses on its molecular biology [18,19], sex pheromones [20], and control methods [21,22], while we lack a systematic understanding of how climate change influences the distribution of *P. interpunctella* in China. Understanding its potential future distribution pattern will enable the governmental pest management agencies to implement corresponding measures to prevent it from continuing to cause losses.

The potential geographical distribution of different invasive species has been shown to be primarily evaluated using ecological niche models, such as ANUCLIM/BIOCLIM, CLIMATE, CLIMEX, DOMAIN, GARP, HABITAT, and MaxEnt [23]. Among these models, CLIMEX is a niche modeling package that was explicitly developed to explore the effects of climate on invasive species and to evaluate their potential distributions in future climates [24]. Insects are cold-blooded species, and climate change within the seasons will have an important impact on insects, such as through changing the spatial organization and cellular membranes of the cytoplasm in insects [25]. The relevant biological parameters of insects may change rapidly after they invade a new area [26]. In CLIMEX, biological data can be updated and modeled repeatedly, which can make the results more accurate. It is primarily applied to destructive species in agricultural production, including injurious insects, weeds, and crop diseases [27,28,29]. The main advantage of CLIMEX is that it can be calibrated theoretically according to geographical distribution data and phenological observations [30].

There is little research on the detailed potential distribution of *P. interpunctella* in China under future climate conditions. We adjusted parameter values according to the published literature to better fit the known distribution in China [31]. In this study, using CLIMEX model software to predict the potential distribution characteristics of *P. interpunctella* in China under historical and future climatic conditions is helpful for relevant pest management departments to implement early warning and prevention policies.

## 2. Materials and Methods

### 2.1. CLIMEX Model

The CLIMEX model is a dynamic and bioclimatic niche simulation model that uses species parameters and climate data to predict the potential geographical distribution of species [32]. In this study, we used the ‘Compare Location’ function of CLIMEX version 4.0 (Hearne Scientific Software, Australia) to conduct an analysis. The model evaluates climatic suitability for a species in a named location by calculating an ecoclimatic index (EI) that theoretically ranges from 0 (unsuitable) to 100 (suitable all year round). As a matter of fact, the climate in most areas is constantly changing, and scores of 100 are hardly achieved, with such scores only being achieved in locations with high climatic stability, such as some equatorial regions. The EI value was calculated according to the annual growth index (GI), the stress index (SI), and the stress interaction index (SX). For additional details on the computational formula, see Kriticos et al. [33].

### 2.2. Climate Data

CliMond (https://www.climond.org/ (accessed on 4 November 2021)) gridded 10’ spatial resolution meteorological data were used for modeling the distribution of *P. interpunctella* in CLIMEX because of its good spatial resolution [34]. Detailed meteorological data included the values of the monthly average minimum and maximum temperatures, rainfall, and relative humidity at 9 a.m. and 3 p.m. The A2 SRES scenario proposed in the fourth assessment report of the Intergovernmental Panel on Climate Change (IPCC) and the global climate model CSIRO-Mk3.0 from the Centre for Climate Research, Australia, were used to model the distribution of *P. interpunctella* in the future climate change scenarios predicted for 2030, 2050, and 2070 [35]. A2 SRES is not only the best equivalence to the recently updated greenhouse gas trajectories, RCP 8.5 (the representative concentration pathway-RCP), but also represents data of technological, demographic, and economic variables related to greenhouse gas (GHG) emissions from independent and self-reliant countries [36,37].

### 2.3. Collection of P. interpunctella Distribution Data in China

Records detailing the distribution of *P. interpunctella* in China were obtained from the Commonwealth Agricultural Bureaux International (CABI), the Global Biodiversity Information Facility (GBIF), and the published literature reviews related to biological characteristics, as well as scientific information on Web of Science, Science Direct, China National Knowledge Internet (CNKI http://cnki.net/ (accessed on 23 December 2021)), PubMed, and ASP. Up until now, the *P. interpunctella* has been distributed in most parts of China, except in areas with a harsh climate.

### 2.4. Fitting CLIMEX Parameters

#### 2.4.1. Temperature Index (TI) and Length of the Growing Season (PDD)

When *P. interpunctella* feeds on different diets, differences can be observed in its biological parameters. Rice is the staple food of most people in China and is stored in every household [38]. China ranks first in rice consumption and output in the world [39], so it was more representative of the damage caused by *P. interpunctella* to rice. Therefore, we chose to set the relevant biological parameters of *P. interpunctella* as the standard when it fed rice. Jung [31] found that when fed rice, the starting temperature (DV0) was 12.5 °C and that the survival rate was only 1% when the culture temperature was 35 °C, and when calculated, it took 461 °C days for the species to develop from an egg to an adult. Studies have confirmed that the survival rate of the *P. interpunctella* is higher when the temperature range is 20–28 °C, with growth inhibition being observed at 32–35 °C [40]. Lee’s experimental data showed that *P. interpunctella* died within 15 d of being at 30 °C [41]. Based on the above biological information, the starting temperature (DV0) was set to 12.5 °C, and lower optimal temperature (DV1) and upper optimal temperature (DV2) were set to 20 °C and 30 °C, respectively. The upper threshold temperature (DV3) was estimated as 35 °C, and the degree day (PDD) was set to 461 °C days.

#### 2.4.2. Moisture Index (MI)

There are few moisture index studies related to *P. interpunctella*. When fresh vegetables and fruits are dried, they emit fragrances and are easily infected by *P. interpunctella*. Therefore, combined with the lowest default value provided by the CLIMEX template, the lower soil moisture threshold (SM0) was set to 0.05 to indicate that *P. interpunctella* can continue to develop in dry environments. The lower optimal soil moisture (SM1) was set to 0.2, representing relatively dry soil, to match the distribution records of *P. interpunctella* in northwest Xinjiang, China. The widespread distribution of *P. interpunctella* in Shanxi, Henan, and other northern provinces shows that it can grow well in temperate climates. Therefore, according to the template in the software and Jung’s research [31], we also adjusted the upper optimal soil moisture (SM2) and upper soil moisture threshold (SM3) to 0.8 and 1.5, respectively.

#### 2.4.3. Stress Index (SI)

Heat Stress: The heat stress threshold temperature (TTHS) was estimated to be the same as DV3 (35 °C), and the heat stress temperature rate (THHS) was estimated to be 0.0048; *P. interpunctella* is unable to survive at temperatures above 35 °C.

Cold Stress: Using information from a supercooling point study of *P. interpunctella* larvae by Feng et al. [42], the cold stress temperature threshold (TTCS) was set to −12.2 °C. The cold stress temperature rate (THCS) was set to −0.0003/week to fit distribution records of *P. interpunctella* in the southwest part of Liaoning province and in the midwest part of Inner Mongolia [43].

Dry Stress and Wet Stress: Due to the lack of available information on the dry and wet stress of *P. interpunctella*, the relevant parameter values could not be estimated. Recent surveys show that the frequency of the *P. interpunctella* in northern China is significantly higher than that in southern China, with the highest frequency in Shanxi Province [44]. Shanxi has a warm temperate continental monsoon climate, so based on the temperate template of ClIMEX, the values for the moisture index and for the dry and wet parameters were adjusted according to Jung et al. [31]. We thus set the dry stress threshold (SMDS) to 0.05, which was consistent with SM0. The wet stress threshold (SMWS) was set to 1.5 in reference to SM3. The accumulation rates for dry stress (HDS) and wet stress (HWS) were set to −0.005 and 0.002, respectively. The parameters that were used are shown in Table 1.

Moisture parameters without units are dimensionless indices of soil moisture (0 = over dry, 1 = field capacity).

In order to present the suitability of *P. interpunctella* in different areas of China more intuitively, we refer to its actual distribution in China and Lee’s research [41] and divide the calculated EI values into four categories: unfavorable habitats (EI = 0), marginally favorable habitats (0 < EI ≤ 10), favorable habitats (10 < EI ≤ 20), and highly favorable habitats (EI > 20).

## 3. Results

### 3.1. Potential Distribution of P. interpunctella under Historical Climate Conditions

**Figure 1:**
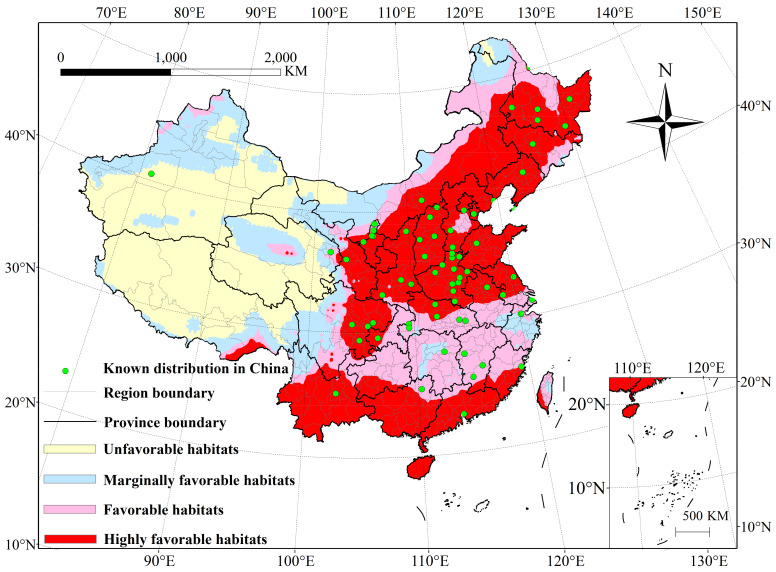
Potential distribution of *P. interpunctella* in China under historical climate conditions (1960–1990).

The model prediction results show that, except for Alar, the recorded distributions collected from CNKI were within the potential distribution area predicted by CLIMEX (Figure 1). This proves that the final parameters can reliably predict the potential distribution of *P. interpunctella* in China. From the distribution map generated by ArcMap, we can calculate the total area of the potential distribution of *P. interpunctella* in China to be 7.00 million km^2^; this represents 72.92% of the total area of mainland China. The area comprising a highly favorable habitat was 3.37 million km^2^, which is 35.11% of the total area of mainland China and accounts for 48.14% of the total area of potential distribution. This potential distribution includes most of the area of Heilongjiang, Jilin, Hebei, Ningxia, Guangdong, Guanxi and Yunnan, Liaoning, Beijing, Tianjin, Shanxi, Shaanxi, Shandong, Henan, Guangdong, Guanxi, Yunnan, the southeast of Gansu, the east Sichuan, the north of Jiangsu and Anhui, the south of Guizhou and Fujian, the northeast of Inner Mongolia, the southwest Taiwan, and a small area of Tibet.

The area of the favorable habitat was 1.78 million km^2^, which is 18.54% of the total area of mainland China and accounts for 25.43% of all the area of potential distribution. The distribution of this habitat covers southern Hubei, Jiangsu, and Anhui; most areas of Hunan and Jiangxi; northern Guizhou, Inner Mongolia, Fujian, and Taiwan; and small areas of Hebei, Heilongjiang, Jilin, Qinghai, Gansu, Yunnan, Guangdong, Guangxi, and Tibet. The area of the marginally favorable habitat was 1.85 million km^2^, which is 19.27% of the total area of mainland China and accounts for 26.43% of the entire region of potential distribution. The main areas belonging to this habitat were the northwest of Gansu, Xingjiang, and Qinghai; the middle part of Hunan, Inner Mongolia, and Zhejiang; northern Yunnan; western Sichuan; and southeastern Tibet.

### 3.2. Potential Distribution of P. interpunctella in Future Climate

**Figure 2:**
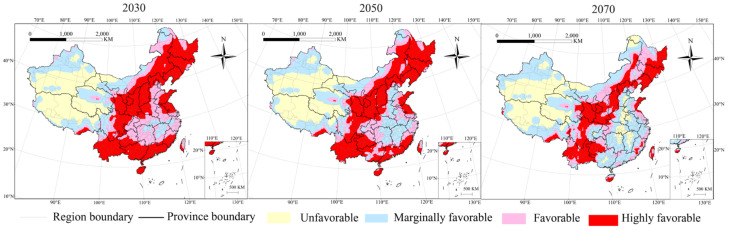
Potential distribution of *P. interpunctella* in China under future climate conditions (2030, 2050, 2070).

The potential distribution range of *P. interpunctella* in China under future climate conditions is shown in Figure 2. The distribution of the highly favorable habitat will decrease to include most provinces. The areas of Guangxi, Guangdong, Fujian, Jiangsu, and Anhui will gradually become marginally favorable habitat areas with increased distribution, as would the eastern parts of Qinghai and Taiwan. For the favorable habitat, the main areas would be located in the northwest of Heilongjiang, Jilin, and Liaoning; northern Shaanxi and Ningxia; northeast Guizhou; the northern and southern areas of Yunnan; central Inner Mongolia; and the coastlines of the coastal provinces. For the marginally favorable habitat, the distribution range in northwest China would remain largely unchanged with areas of increased distribution, including Guangdong, Guangxi, Fujian, and Ningxia. The southern areas of Hebei, northern Hunan and Jiangxi, southeast Hebei, and mid-east part of Hubei would gradually become unfavorable habitat areas.

**Figure 3:**
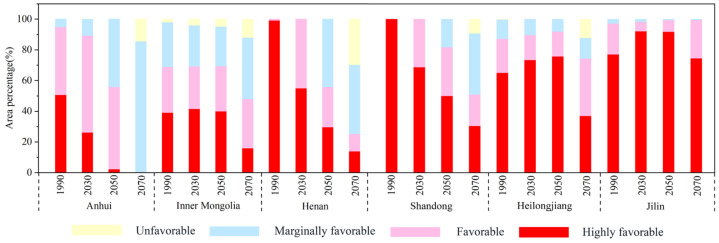
Area percentage changes in different ranges for the suitable distribution for *P. interpunctella* under current and future climate conditions in six major agricultural provinces of China.

**Figure 4:**
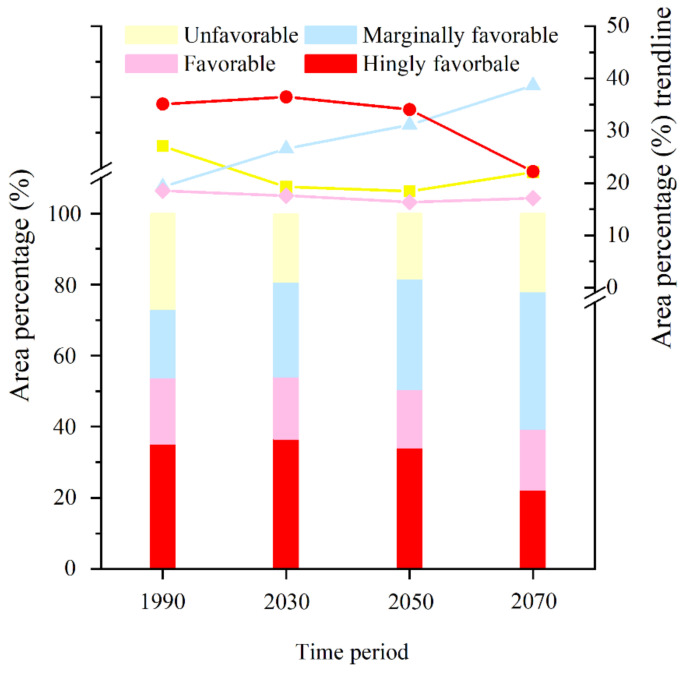
The percentages of various habitats during different periods and their trendlines in the total distribution area of *P. interpunctella* in China.

Figure 3 shows the distribution of potentially suitable areas for *P. interpunctella* in the six major agricultural provinces of China [45]. Before 2050, these six provinces were suitable for the survival of *P. interpunctella* for a long time. By 2070, except for Jilin, the unfavorable habitat areas in five other provinces had begun to increase. The highly favorable habitat areas of Anhui, Henan, and Shandong decrease year by year, and those of Inner Mongolia, Heilongjiang, and Jinlin increase and then decline. Figure 4 shows changes in the suitable distribution area of *P. interpunctella* in China over time. The total region of potential distribution and the area covered by marginally favorable habitat will increase, while the district covered by highly favorable habitat will show a marked decline, with the favorable habitat changing very little. The area of the total potential distribution is observed to increase by 480,000 km^2^ from 1990 to 2070. The increased region of marginally favorable habitat is the largest, covering an area of 1.86 million km^2^. The reduced area of the highly favorable habitat was 1.24 million km^2^.

### 3.3. Change in EI Value on the Potential Distribution under Future Climate Conditions

**Figure 5:**
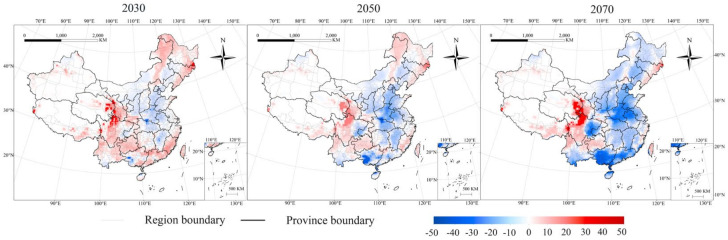
The change in the EI values on interpolation maps for *P. interpunctella* under current and future climate conditions. Red represents the increase in EI values and blue indicates the decrease in EI values. The color becomes darker as the difference in the EI value increases.

**Figure 6:**
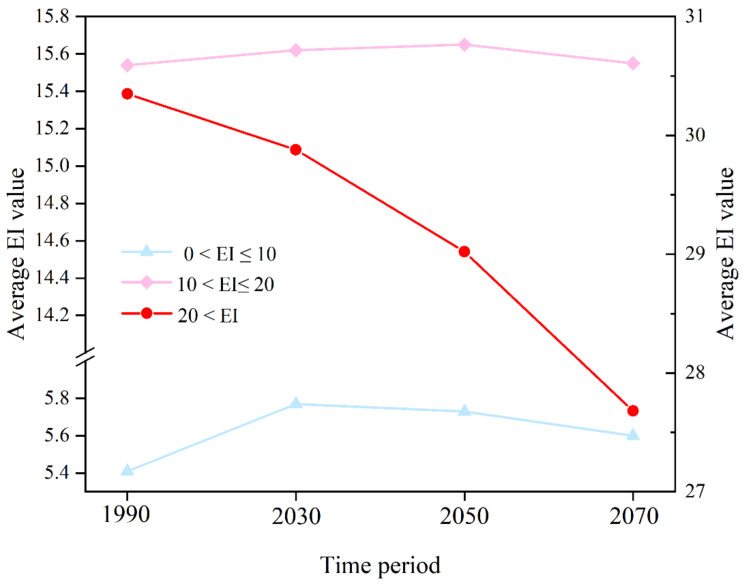
Average EI values of the different potential distributions of habitats under different periods. (right Y-axes: red = highly favorable habitats; upper left Y-axes: pink = favorable habitat; bottom left Y-axes: blue = marginally favorable habitats).

The subtraction of the difference data between three sets of future climate EI values and historical climate EI values was interpolated into ArcMap, generating EI interpolation variation maps (Figure 5). We found that climate change has a great influence on the average EI value of highly favorable habitats (Figure 6).

By comparing the changes favoring *P. interpunctella* survival in China during different periods from 2030 to 2070, the climatic favorability levels increase in the southeast of Liaoning, Tibet, and Jilin; the northern regions of Yunnan; the southern regions of Sichuan and Gansu; and most areas of Guizhou and Taiwan, and the EI value increased from 1 to 40. The EI values for the north of Sichuan; the eastern part of Qinghai; and the junction of Gansu, Qinghai, and Sichuan would increase the most, by up to 40. Climatic favorability levels for *P. interpunctella* would decrease in the northern, southern, and east-central parts of China, with decreasing EI values ranging from 1–32. EI values for the southwest of Guangdong, most of Guangxi, and the northern part of Hainan would decrease the most, reaching 42.

## 4. Discussion

The development of models for predicting the potential distribution of insects in different climatic conditions makes the prediction results more accurate and can provide valuable data for the monitoring and control of harmful insects [30,46]. CLIMEX can model environmental suitability for a species on a seasonal basis. It has been used to predict the distributions of many species of insects [47]. *P. interpunctella* has the characteristics of euryphagy and crypticity, and it is distributed all over the world, which indicates that it has a strong continuous transmission ability. Therefore, we predicted its potential distribution under the current and future A2 SRES climate scenarios in China. Among the cities in China where the distribution of *P. interpunctella* is known, except for Alar, Xinjiang, the rest are within our forecast range, which proves that our model is reliable.

The predicted climate changes will affect *P. interpunctella* negatively in the central-eastern and southern provinces of China. In general, the total area suitable for this pest will decrease by 2070. It should also be noted that northwestern China will become more suitable. The global climate model (GCM) CSIRO-Mk3.0 (CS) data under the A2 SRES scenario used in this study predict that the global climate is warming at a rate of 0.5 °C per decade and that precipitation will decrease [37]. Previous studies have shown that *P. interpunctella* cannot survive above 35 °C and that high temperatures inhibit its growth, with a survival rate of only 1% when the temperature was 35 °C [12,31,48]. In the south of the Yangtze River in China, the number of days when the temperature reaches 35 °C or above can reach 20–30 days [49]. By 2070, the increase of 2.5°C may increase insect mortality under heat and humidity stress. However, in the western region, because of the decrease in cold stress caused by the increase in temperature, this region will be more suitable for the growth of *P. interpunctella*.

Before this study, Jung et al. [31] predicted the potential distribution of *P. interpunctella* around the world according to four different diets. Jung indicated that rice production areas, such as Hunan and Jiangxi, are high-risk areas that are susceptible to infestation by the *P. interpunctella*, and our findings support this conclusion. Jung predicted that only Henan province in northern China has EI values that indicate that it could be infested by the *P. interpunctella*, while our model showed that most of the provinces in northern China had high EI values. The report shows that, among the different grain-producing areas of the 11 provinces in China’s main grain storage ecological areas, the occurrence frequency of *P. interpunctella* is the highest in Shanxi, Liaoning, and Henan in the north, with occurrence frequencies of 42.3%, 39.5%, and 36.8%, respectively [44], which proves that the prediction results of our model conform more to the actual distribution of *P. interpunctella* in China. Because the climate data are from the same source, the differences in the biological parameter settings lead to differences between the two research results. In recent years, the discovery sites of *P. interpunctella* in China have continued to increase. Based on its biological parameters, such as its supercooling point and current distribution, the parameters have been adjusted. Finally, the EI values calculated by the model may be different, which makes the specific prediction distribution range different.

China’s terrain has gradually increased from the eastern coast to the western inland area and is arranged in stepped shape [50]. The average level of altitude is between 500 m to 4000 m, and the differences between provinces are significant. Among them, the Qinghai–Tibet region has the highest average altitude, the main terrain of which is the Qinghai–Tibet Plateau, which is the largest plateau in China and the highest in the world and is also known as the “Third Pole” [51]. In this area, the long winters are dry and cold, and the radiation is strong, making it difficult for *P. interpunctella* to survive under such conditions. The Tarim Basin in the south of Xinjiang has a higher distribution of desert, low vegetation coverage, perennial natural disasters, such as sandstorms and dry–hot wind, and a high dry stress index, which is also not suitable for the growth of *P. interpunctella*. In recent years, the Zhungeer basin in the north of Xinjiang and the Qaidam basin in the northwest of Qinghai have experienced changes in their local hydrothermal conditions due to the continuous transformation of the local agricultural ecosystem [52]. Both have oasis areas suitable for farming, which can produce various kinds of food and are potential distribution areas of *P. interpunctella*. The plain in the northern and hilly areas in the south is mild and suitable for planting, and most areas are in a highly suitable living environment for *P. interpunctella*, making it extremely vulnerable to infection.

CLIMEX model predictions are primarily based on climatic factors, but nonclimatic factors, such as biological interactions, genetic evolution, graphic barriers, and human activities, might affect the studies of species distribution [53]. By monitoring storage facilities, Zhao found that the external temperature of the granary gradually increased with the outer grain temperature also rising gradually, while the interior of the grain pile was still maintaining a lower temperature. There is a warm airflow inside the granary, which gradually penetrates into the interior of the grain pile, and a high-humidity area is readily generated at the junction of the cold and warm airflow, 300 to 500 mm from the surface of the grain pile. The difference between the maximum and minimum temperatures inside the storage room also have been shown to be less than that outside [54]. At the same time, the research also shows that the difference between the maximum and minimum temperatures in the storage facility is smaller than that outside [55]. However, in developing countries, such as China, after harvesting grains, farmers usually store them in jute bags, propylene sacks, or traditional cribs, and many areas, especially remote rural areas, do not have storage facility with good conditions; the temperature of grains basically varies with the outside world [56]. When grain is infected by pests, the heat and moisture produced by the metabolism of feeding larvae will also lead to the increase in grain temperature [57]. The temperature and humidity in the granary are closely related to the activity of pests [58], and their constant changes may change the biological parameters of pests. When *P. interpunctella* harms stored grain, its biological characteristics will change with time due to the different positions and environments of granaries, and these factors may also affect the predicted distribution. The adults of *P. interpunctella* have strong mobility, which can infect storage within the range of 21~276 m, and its dispersion ability also has an important influence on the distribution [59]. Recent studies have shown that genetic variation and climate models can be combined to predict changes in the range of invasive pests under future climates [60]. With the development and progress of species distribution models, the influence of nonclimatic factors on the potential distribution of *P. interpunctella* will be further explored. In a subsequent study, we will also continue to investigate the distribution of the *P. interpunctella* in China, explore the specific effects of external environmental changes in the biological parameters of the *P. interpunctella*, and establish a model evaluation system to improve model performance.

The model in this study shows that in the next 50 years, most of China will be a potentially suitable distribution area for *P. interpunctella*. Since the beginning of the 21st century, grain production in northern China has increased substantially and has been shipped to the south in large quantities, directly promoting the construction of grain logistics node layout, logistics parks, and grain storage warehouses [61]. The warehouse is not only stable in the environment but also abundant in food. In addition, the larvae and eggs of *P. interpunctella* are small, so it is easy for it to spread with the host for a long period time. If no active control measures are taken, the spread of *P. interpunctella* in China will continue to accelerate. The intelligent detection and early warning technology of *P. interpunctella* can determine its dynamic changes in a timely manner and accurately provide a powerful guarantee for preventing it from infecting stored grains. Near-infrared spectroscopy (NIR) coupled with discriminant and modeling classification methods can be used to detect the presence of *P. interpunctella* and is not destructive to the tested grains [62]. Metal silos have been proven to be effective in protecting stored grains and in killing any insect pests that may be present [63]. Once grain is found to be infected by *P. interpunctella*, it can be used to control gamma radiation, gaseous ozone, and high nitrogen [64,65,66]. Of course, biological control can also be performed by releasing parasitoids [67]. The chemical fumigants methyl bromide and phosphine are usually used to control *P. interpunctella* in large commodities. However, the extensive use of methyl bromide has been proven to cause damage to the ozone layer, and it is being phased out [68,69]. Phosphine has an obvious insecticide resistance problem, and its control effect is gradually weakened [70]. Studies have confirmed that climate warming not only expands the overwintering range of global pests but also promotes the emergence of insect resistance [71]. Extracting naturally occurring toxins from plant essential oils and developing them into new pesticides is a future research direction because they are usually safe and fully biodegradable [72].

## 5. Conclusions

In this study, we analyzed the potential distribution of *P. interpunctella* in China under historical and future climate data using the CLIMEX model. The results show that in the next 50 years, most areas of China are potential distribution areas for *P. interpunctella,* except for parts of the Qinghai–Tibet Plateau and Tarim Basin. With the warming of the climate, the expanding areas are mainly located in the eastern part of Qinghai, while the mid-eastern, northeastern, and southeastern parts of China appear likely to become unfavorable areas. China should continue to implement strict monitoring, prevention, and control measures for *P. interpunctella* to prevent its spread all over the country and to achieve the goal of ensuring food security.

## Figures and Tables

**Table 1:** CLIMEX parameter values used for *P. interpunctella* modelling.

CLIMEX Parameters		Values	Unit
Temperature			
DV0	Starting temperature	12.5	°C
DV1	Lower optimal temperature	20	°C
DV2	Upper optimal temperature	30	°C
DV3	Upper temperature threshold	35	°C
PDD	Degree days to complete one generation	461	°C days
Moisture			
SM0	Lower soil moisture threshold	0.05	
SM1	Lower optimal soil moisture	0.2	
SM2	Upper optimal soil moisture	0.8	
SM3	Upper soil moisture threshold	1.5	
Heat stress			
TTHS	Heat stress temperature threshold	35	°C
THHS	Heat stress temperature rate	0.0048	Week^−1^
Cold stress			
TTCS	Cold stress temperature threshold	−12.2	°C
THCS	Cold stress temperature rate	−0.0003	Week^−1^
Dry stress			
SMDS	Dry stress threshold	0.05	
HDS	Dry stress rate	−0.005	Week^−1^
Wet stress			
SMWS	Wet stress threshold	1.5	
HWS	Wet stress rate	0.002	Week^−1^

## Data Availability

The data presented in this study are available in article materials.
